# The impact of the COVID-19 pandemic on access to supervised consumption programs

**DOI:** 10.1186/s13011-023-00521-6

**Published:** 2023-03-10

**Authors:** Emili Gubskaya, Mary Clare Kennedy, Kanna Hayashi, Zishan Cui, M-J Milloy, Thomas Kerr

**Affiliations:** 1 British Columbia Centre on Substance Use, 400-1045 Howe Street, Vancouver, BC V6Z 2A9 Canada; 2 Department of Medicine, University of British Columbia, St. Paul’s Hospital, 608-1081 Burrard Street, Vancouver, BC V6Z 1Y6 Canada; 3 School of Social Work, University of British Columbia Okanagan, 1147 Research Road, Kelowna, BC V1V 1V7 Canada; 4 Faculty of Health Sciences, Simon Fraser University, 888 University Drive, Burnaby, BC V5A 1S6 Canada

**Keywords:** COVID-19, Supervised consumption sites, Opioid, Overdose, Access, People who use drugs, Public health, Harm reduction

## Abstract

**Background:**

Supervised consumption sites (SCS) and overdose prevention sites (OPS) have been increasingly implemented in response to the ongoing overdose epidemic in Canada. Although there has been a dramatic increase in overdose deaths since the start of the SARS-CoV 2 (COVID-19) pandemic, little is known about how SCS access may have been affected by this pandemic. Therefore, we sought to characterize potential changes in access to SCS during the COVID-19 pandemic among people who use drugs (PWUD) in Vancouver, Canada.

**Methods:**

Between June and December 2020, data were collected through the Vancouver Injection Drug Users Study (VIDUS) and the AIDS Care Cohort to Evaluate Exposure to Survival Services (ACCESS), two cohort studies involving people who use drugs. Multivariable logistic regression was used to examine individual, social and structural factors associated with self-reported reduced frequency of SCS/OPS use since COVID-19.

**Results:**

Among 428 participants, 223 (54.7%) self-identified as male. Among all individuals surveyed, 63 (14.8%) reported a decreased frequency of use of SCS/OPS since COVID-19. However, 281 (66%) reported that they “did not want to” access SCS in the last 6 months. In multivariable analyses, younger age, self-reported fentanyl contamination of drugs used and reduced ease of access to SCS/OPS since COVID-19 were positively associated with a decreased frequency of use of SCS/OPS since COVID-19 (all *p* < 0.05).

**Conclusions:**

Approximately 15% of PWUD who accessed SCS/OPS reported reduced use of these programs during the COVID-19 pandemic, including those at heightened risk of overdose due to fentanyl exposure. Given the ongoing overdose epidemic, efforts must be made to remove barriers to SCS access throughout public health crises.

## Background

North America remains in the midst of an escalating overdose epidemic driven largely by the proliferation of synthetic opioids in the illicit drug supply. Unfortunately, since the start of the SARS-CoV 2 (COVID-19) pandemic, overdose-related deaths have increased dramatically. In Canada, opioid toxicity deaths increased from 3747 (April 2019 – March 2020) to 7362 (April 2020 – March 2021), an increase of nearly 96% [10, 15].

Prior to the pandemic supervised consumption sites (SCS) were one of the many strategies being employed in Canada to address the overdose crisis [9, 26]. SCS, also known as safe injection sites or overdose prevention sites (OPS), are places where people who use drugs (PWUD) can go to safely consume pre-obtained substances under supervision [3]. SCS staff also offer life-saving support in the event of overdose and referrals to external services [17]. As of January 2020, more than 70 SCS were operating in Canada, the overwhelming majority of which opened after 2017. Of these, 9 SCS were operating in Vancouver [16].

SCS have been shown to provide a number of health benefits among PWUD [11], including reduced risk of overdose mortality. For example, a previous study found that after the opening of the first SCS in Vancouver, overdose-related deaths decreased by 35% in city blocks within 500 m of the SCS [13]. Another comprehensive review of evidence derived from SCS evaluations noted that there had been no overdose deaths in any SCS to date as SCS are well equipped to handle overdose incidents [18].

With the emergence of the COVID-19 pandemic, SCS in many settings in Canada and elsewhere experienced disruptions in service delivery, including closures, difficulty communicating with clients about service changes, and restrictions on capacity [4]. In British Columbia, monthly SCS visits dropped from nearly 60, 000 per month to approximately 23, 000 per month [1]. A qualitative study done in Canada found that 53% of PWUD who also used harm reduction services in their sample population felt that there were negative changes in service delivery. They further go on to report that public health measures implemented in response to COVID-19 further negatively impacted PWUD. Some of these impacts include increased substance use, sharing/re-use of substance supplies, unattended overdose events, and food and housing insecurity [20].

However, we know of few studies that have examined potential changes in SCS utilization patterns among PWUD after the emergence of the pandemic, including decreased frequency of use of these services. We therefore sought to characterize decreased frequency of SCS use after the declaration of the COVID-19 pandemic as a public health emergency among PWUD participating in two prospective cohort studies in Vancouver, Canada. Understanding these changes will allow us to further inform efforts to ensure functional access to harm reduction services throughout public health crises.

## Methods

Data for this study were drawn from two open prospective cohort studies of PWUD in Vancouver, Canada: the Vancouver Injection Drug Users Study (VIDUS) and the AIDS Care Cohort to evaluate Exposure to Survival Services (ACCESS). Both cohorts have been described in detail in previous literature [24, 27]. However, to briefly summarize, these cohorts have been recruiting participants through community-based methods, including street outreach, self-referral, and word of mouth since May of 1996. VIDUS includes adults (18 years and older) who are HIV-negative and have injected unregulated drugs within the month prior to their enrolment. ACCESS participants are HIV-positive adults who used any unregulated substance (other than or in addition to cannabis) within the month prior to their enrolment. Participants in the VIDUS cohort who HIV seroconvert after their enrolment are transferred to the ACCESS cohort. All participants provided written informed consent at enrolment and ethics has been approved by Providence Health Care/University of British Columbia’s Research Ethics Board. Both cohorts use harmonized study protocols to facilitate pooled analyses.

At baseline and at 6-month intervals afterwards, participants complete interviewer- and nurse-led questionnaires and provide blood samples for serology, as well as urine for drug screening. The questionnaire covers a variety of topics including demographics, substance use, healthcare access, and socio-structural exposures. To compensate participants for their involvement, participants receive a $40 CAD stipend for every study visit.

Due to the COVID-19 pandemic, all in-person data collection was suspended between March 2020 and July 2020. After July 2020, infection control measures were put in place to resume data collection. Participant interviews were completed over telephone or videoconferencing. Study-owned cell phones and private spaces were loaned to those who required them. They were then able to pick up their cash honoraria in person or have it e-transferred if they had access to a bank account.

Between March and July of 2020, study questionnaires were modified to include questions regarding the COVID-19 pandemic. One of these questions was used to assess the primary outcome of this study, which read as follows: “Has the frequency of your use of these sites [i.e., SCS/OPS] changed since the beginning of the public health emergency?”. The outcome was dichotomized using the following responses: “I use them less” vs. “I use them more” or “My use stayed the same”. Potential correlates were identified based on past studies that assessed SCS access among PWUD [8, 25], and included: age (per year older), self-identified gender (man vs. woman/other), ethnicity/ancestry (white vs. Black, Indigenous, and people of colour), education (high school or greater vs. other), employment (yes vs. no), residence in Downtown Eastside neighbourhood in Vancouver (yes vs. no), daily non-medical prescription opioid use (yes vs. no), daily cocaine use (yes vs. no), daily crystal methamphetamine use (yes vs. no), daily non-injection crack-cocaine use (yes vs. no), benzodiazepine use (yes vs. no), suspected that a drug used contained fentanyl (yes vs. no), used drugs alone (yes vs. no), engagement in opioid agonist therapy (yes vs. no), non-fatal overdose (yes vs. no), witnessed an overdose (yes vs. no), experience physical violence (yes vs. no), syringe/ drug use equipment sharing (yes vs. no), inability to access treatment (yes vs. no), unstable housing (yes vs. no), sex work (yes vs. no), incarceration (yes vs. no), jacked up (this refers to being stopped, searched, or detained) by the police (yes vs. no), cohort/ HIV status (ACCESS vs. VIDUS), ever tested positive for COVID-19 (yes vs. no), concern about COVID-19 on a scale from 1 to 10, with 10 indicating greatest concern (1–5 vs. 6–10), any chronic health conditions (yes vs. no), and ease of accessing SCS/OPS changed since COVID-19 (same vs. easier vs. harder). All drug use and behavioral variables refer to the 6 months prior to questionnaire date unless otherwise indicated.

Univariable and multivariable logistic regression analyses were used to assess the associations between the correlates of interest and reduced frequency of SCS/OPS use since COVID-19. Correlates of interest with a univariable *p*-value < 0.10 were included in a backward elimination procedure, with the least significant variable removed at each step until the lowest Akaike Information Criterion (AIC) was achieved. All *p*-values were two-sided and all statistical analyses were conducted using SAS version 9.4 (SAS Institute, Cary, North Carolina, United States).

## Results

In total, there were 428 people who use drugs included in this analysis, of which 223 (54.7%) were male. The median age was 51 years old (1st to 3rd quartile = 42–58). Among all individuals surveyed, 63 (14.8%) reported a decreased frequency of use of SCS/OPS since COVID-19. However, when asked about accessing SCS, 281 of all individuals reported that they “did not want to” in the last 6 months. In univariable logistic regression analyses (Table 1), factors positively associated with a decreased frequency of SCS/OPS use since COVID-19 include younger age (Odds ratio [OR] = 0.95, 95% confidence interval [CI]: 0.92–0.98), suspected fentanyl exposure (OR = 4.09, 95% CI: 1.81–9.24), witnessing an overdose (OR = 1.78, 95% CI: 1.01–3.14), unstable housing (OR = 2.04, 95% CI: 1.15–3.36), and decreased ease of access to SCS/OPS since COVID-19 (OR = 6.77, 95% CI: 3.66–12.53).

**Table 1:** Bivariable analyses of factors associated with a decrease in frequency of use of SCS/OPS since COVID-19 among people who use drugs in Vancouver (*n* = 428)

Frequency of SCS/OPS use since COVID-19 pandemic				
Characteristic	Less n (%) 63 (14.8%)	More/Same n (%) 364 (85.3%)	Odds Ratio (95% CI)	***p –*** value
**Age (median, IQR)**	45 (39–53)	52 (43–58)	0.95 (0.92–0.98)	0.000
**Self-identified Gender**				
Female/Other	24 (45.3)	160 (45.2)	1.00 (0.54–1.78)	0.991
Male	29 (54.7)	194 (54.8)		
**Ethnicity/Ancestry**				
White	30 (56.6)	191 (54.3)	0.91 (0.51–1.63)	0.750
BIPOC^**b**^	23 (43.4)	161 (45.7)		
**Education**				
Other	24 (45.3)	182 (52.3)	1.32 (0.74–2.37)	0.341
Highschool or greater	29 (54.7)	166 (47.7)		
**Employment**				
Yes	21 (33.3)	124 (34.1)	0.97 (0.55–1.71)	0.910
No	42 (66.7)	240 (65.9)		
**DTES Residence**^**a**^				
Yes	39 (61.9)	219 (60.2)	1.08 (0.62–1.87)	0.794
No	24 (38.1)	145 (39.8)		
**Daily Rx Opioids Use**^**a**^				
Yes	3 (4.8)	6 (1.7)	2.98 (0.72–12.22)	0.113
No	60 (95.2)	357 (98.4)		
**Daily Cocaine Use**^**a**^				
Yes	1 (1.6)	12 (3.3)	0.47 (0.06–3.70)	0.466
No	62 (98.4)	352 (96.7)		
**Daily Crystal Meth Use**^**a**^				
Yes	13 (20.6)	59 (16.2)	1.34 (0.69–2.63)	0.386
No	50 (79.4)	305 (83.8)		
**Daily Crack Non-Injection**^**a**^				
Yes	6 (9.5)	54 (14.8)	0.60 (0.25–1.47)	0.263
No	57 (90.5)	310 (85.2)		
**Benzodiazepine use**^**a**^				
Yes	4 (6.4)	10 (2.8)	2.40 (0.73–7.90)	0.138
No	59 (93.7)	354 (97.3)		
**Suspected Fentanyl Exposure**^**a**^				
Yes	56 (88.9)	233 (66.2)	4.09 (1.81–9.24)	0.000
No	7 (11.1)	119 (33.8)		
**Used Drug Alone**^**a**^				
Yes	46 (82.1)	187 (79.9)	1.16 (0.54–2.46)	0.706
No	10 (17.9)	47 (20.1)		
**OAT**^**ac**^				
Yes	48 (76.2)	232 (64.1)	1.79 (0.97–3.33)	0.062
No	15 (23.8)	130 (35.9)		
**Non-fatal Overdose**^**a**^				
Yes	12 (19.1)	64 (17.7)	1.10 (0.55–2.17)	0.794
No	51 (81.0)	298 (82.3)		
**Witnessed Overdose**^**a**^				
Yes	40 (65.6)	186 (51.7)	1.78 (1.01–3.14)	0.044
No	21 (34.4)	174 (48.3)		
**Experienced Physical Violence**				
Yes	12 (19.1)	50 (13.7)	1.48 (0.74–2.96)	0.269
No	51 (81.0)	314 (86.3)		
**Equipment Sharing**^**a**^				
Yes	17 (27.9)	82 (23.6)	1.25 (0.68–2.30)	0.476
No	44 (72.1)	265 (76.4)		
**Inability to access treatment**^**a**^				
Yes	1 (1.6)	6 (1.7)	0.99 (0.12–8.36)	0.992
No	60 (98.4)	356 (98.3)		
**Unstable Housing**^**a**^				
Yes	44 (69.8)	192 (53.2)	2.04 (1.15–3.63)	0.014
No	19 (30.2)	169 (46.8)		
**Sex work**^**a**^				
Yes	4 (6.5)	43 (11.8)	0.51 (0.18–1.49)	0.213
No	58 (93.6)	321 (88.2)		
**Incarceration**^**a**^				
Yes	3 (4.8)	8 (2.2)	2.21 (0.57–8.58)	0.239
No	60 (95.2)	354 (97.8)		
**Jacked up**^**a**^				
Yes	4 (6.4)	15 (4.2)	1.56 (0.50–4.86)	0.440
No	59 (93.7)	345 (95.8)		
**Cohort**				
VIDUS	39 (61.9)	198 (54.4)	1.36 (0.79–2.36)	0.268
ACCESS	24 (38.1)	166 (45.6)		
**Believe Infected with Covid**				
Yes	5 (8.1)	22 (6.2)	1.34 (0.49–3.68)	0.596
No	57 (91.9)	336 (93.9)		
**Concern about COVID**				
1–5	27 (43.6)	179 (49.6)	0.78 (0.46–1.35)	0.380
6–10	35 (56.5)	182 (50.4)		
**Any chronic health conditions**				
Yes	52 (82.5)	303 (83.9)	0.90 (0.45–1.84)	0.782
No	11 (17.5)	58 (16.1)		
**Ease of Accessing SCS/OPS changed since COVID-19**				
Harder	19 (49.2)	37 (12.5)	6.77 (3.66–12.53)	< 0.001
Same/Easier	30 (50.9)	259 (87.5)		

Unless otherwise specified, drug use includes injection and non-injection consumption

^a^Denotes behaviours/exposures in the past 6 months

^b^*BIPOC* Black, Indigenous, or other people of colour

^c^*OAT* Opioid agonist therapy

In a multivariable logistic regression analysis (Table 2), factors that remained positively associated with difficulty accessing SCS/OPS included younger age (adjusted odds ratio [AOR] = 0.95, 95% CI: 0.92–0.98), suspected fentanyl exposure (AOR = 2.61, 95% CI: 1.10–6.19) and decreased ease of access to SCS/OPS since COVID-19 (AOR = 5.11, 95% CI: 2.92–10.41).

**Table 2:** Multivariable logistic regression analysis of factors associated with a decrease in frequency of use of SCS/OPS since COVID-19 among people who use drugs in Vancouver (*n* = 428)

Variable	Adjusted Odds Ratio (AOR)	95% Confidence Interval (CI)	***P -*** value
**Age**	0.97	(0.94–1.00)	0.033
**Suspect Drug Contained Fentanyl**^**a**^ (Yes vs. No)	2.61	(1.00–6.19)	0.029
**Ease of Accessing SCS/OPS changed since COVID-19** (Harder vs. Same/Easier)	5.51	(2.92–10.41)	< 0.0001

^a^Denotes behaviours/exposures in the past 6 months

## Discussion

We found that approximately 15% of a community-recruited sample of PWUD in Vancouver reduced their frequency of SCS use after the emergence of the COVID-19 pandemic. In multivariable analyses (Table 2), reduced use of SCS was associated with suspected fentanyl contamination of drugs used and perceived decreased ease of access to SCS/OPS since the onset of restrictions associated with COVID-19.

Our finding that reduced use of SCS occurred among 15% of all PWUD in this study and was associated with perceived decreased ease of SCS accessibility are aligned with other research indicating that the accessibility of many services was compromised during the COVID-19 pandemic [6, 22]. Harm reduction services were no exception [23]. Worldwide, the uncertainty regarding COVID-19 restrictions, as well as challenges associated with efforts to reduce COVID-19 spread while still preventing overdoses, may have contributed to adverse impacts on the accessibility of overdose prevention services, including SCS [7, 19]. Although not explored in this study, factors known to constrain other health programs during the COVID-19 pandemic that may have affected SCS delivery, include concerns among staff about safety, staff shortages, lack of proper access to adequate personal protective equipment (PPE), unforeseen illness, and the rapidly changing global situation [5, 12, 21], which in turn may have prompted changes in the way SCS operate.

While it is evident that COVID-19 public health policies impact the delivery of harm reduction services, our study reveals that these changes had a substantial impact on the access patterns of PWUD in Vancouver. In total, 15% of all PWUD interviewed in our sample reported a decreased frequency of use of SCS/OPS since COVID-19. This is in line with a recent Canadian study which showed that 53% of their participants who used harm reduction services identified negative changes in service delivery [20].

We found that suspected exposure to illicit fentanyl was positively correlated with a reduced frequency of SCS use. Recent data released from the BC Coroner’s service indicates that the proportion of overdose deaths attributable to exposure to high concentrations of fentanyl has increased [2]. This finding stands in contrast to a study conducted in Baltimore prior to the pandemic, which found that “thinking drugs contained fentanyl” was independently associated with willingness to use SCS [14]. Further research should be undertaken to further explore the association observed herein. However, given that SCS are uniquely well-positioned to respond to fentanyl-related overdose, it is concerning that COVID restrictions have created additional obstacles for accessing SCS among those with suspected fentanyl exposure. Accordingly, efforts should be made to minimize barriers to SCS access and increase the availability of services specifically for those at risk of fentanyl exposure.

Our study sheds light on the associations between COVID-19 and access to SCS, and serves to identify subpopulations of PWUD who may be vulnerable to disruptions in service access, including younger individuals and those who report reduced ease of service access. Our findings further suggest that it is possible that reduced SCS access may be a contributing factor to the rise in overdose in recent years. Our study also has limitations. One limitation is that we examined the impacts of COVID-19 on service access at a particular time early in the epidemic, and mitigation strategies have likely evolved since this time. Still, our findings point to the need to continue to find creative ways to improve service access, even when epidemics such as COVID-19 are occurring. Another limitation is that the cohort studies included in this analysis are not randomly recruited and therefore may not be representative of PWUD in Vancouver or elsewhere. Further, we relied on self-report, which may be vulnerable to socially desirable responding and recall bias. Given the cross-sectional nature of this study, we cannot infer causation, and unmeasured confounding may be present.

## Conclusion

In summary, a small but significant proportion of participants in our study reported reduced use of SCS in Vancouver during the COVID-19 pandemic, with younger participants, as well as those who reported suspected fentanyl exposure and reduced ease of SCS access being more likely to report reduced use of SCS. Given the ongoing overdose epidemic, it is therefore critical that efforts are made to ensure accessible harm reduction services, including during concordant public health crises.

## Data Availability

The data used for this study are not publicly available and can be requested from the corresponding author on reasonable request and with permission of the University of British Columbia/Providence Health Care Research Ethics Board.

## Abbreviations

SCS: Safe Consumption Site
OPS: Overdose Prevention Site
PWUD: People Who Use Drugs
COVID-19: SARS-CoV 2
VIDUS: Vancouver Injection Drug Users Study
ACCESS: AIDS Care Cohort to Evaluate Exposure to Survival Services
BC: British Columbia
OR: Odds Ratio
CI: Confidence Interval
AIC: Akaike Information Criterion
